# Uncovering the role of TET2-mediated ENPEP activation in trophoblast cell fate determination

**DOI:** 10.1007/s00018-024-05306-z

**Published:** 2024-06-17

**Authors:** Wen Huang, Andy Chun Hang Chen, Xujin Wei, Sze Wan Fong, William Shu Biu Yeung, Yin Lau Lee

**Affiliations:** 1 Department of Obstetrics and Gynaecology, Li Ka Shing Faculty of Medicine, School of Clinical Medicine, The University of Hong Kong, Hong Kong, Special Administrative Region China; 2 Centre for Translational Stem Cell Biology, Science Park, Sha Tin , Hong Kong, Special Administrative Region China; 3 Shenzhen Key Laboratory of Fertility Regulation, Reproductive Medicine Center, The University of Hong Kong-Shenzhen Hospital, Shenzhen, China

**Keywords:** Early trophoblast development, Human expanded potential stem cells, DNA methylation, Ten-eleven translocation methylcytosine dioxygenase 2, Glutamyl aminopeptidase

## Abstract

Early trophoblast differentiation is crucial for embryo implantation, placentation and fetal development. Dynamic changes in DNA methylation occur during preimplantation development and are critical for cell fate determination. However, the underlying regulatory mechanism remains unclear. Recently, we derived morula-like expanded potential stem cells from human preimplantation embryos (hEPSC-em), providing a valuable tool for studying early trophoblast differentiation. Data analysis on published datasets showed differential expressions of DNA methylation enzymes during early trophoblast differentiation in human embryos and hEPSC-em derived trophoblastic spheroids. We demonstrated downregulation of DNA methyltransferase 3 members (*DNMT3s*) and upregulation of ten-eleven translocation methylcytosine dioxygenases (*TETs*) during trophoblast differentiation. While DNMT inhibitor promoted trophoblast differentiation, TET inhibitor hindered the process and reduced implantation potential of trophoblastic spheroids. Further integrative analysis identified that glutamyl aminopeptidase (ENPEP), a trophectoderm progenitor marker, was hypomethylated and highly expressed in trophoblast lineages. Concordantly, progressive loss of DNA methylation in *ENPEP* promoter and increased ENPEP expression were detected in trophoblast differentiation. Knockout of *ENPEP* in hEPSC-em compromised trophoblast differentiation potency, reduced adhesion and invasion of trophoblastic spheroids, and impeded trophoblastic stem cell (TSC) derivation. Importantly, TET2 was involved in the loss of DNA methylation and activation of *ENPEP* expression during trophoblast differentiation. *TET2*-null hEPSC-em failed to produce TSC properly. Collectively, our results illustrated the crucial roles of ENPEP and TET2 in trophoblast fate commitments and the unprecedented TET2-mediated loss of DNA methylation in *ENPEP* promoter.

**Supplementary Information:**

The online version contains supplementary material available at 10.1007/s00018-024-05306-z.

## Introduction

The first lineage segregation of human preimplantation embryos involves the formation of trophectoderm (TE) and inner cell mass (ICM) [1]. While the ICM further develops into the embryo proper, TE differentiates into all the lineages of the placenta, including cytotrophoblasts (CTB), syncytiotrophoblasts (STB) and extra-villous trophoblasts (EVT). The early trophoblasts are essential for a successful pregnancy by controlling the implantation, hormone production and immune protection of the fetus [2]. The mechanisms regulating the first lineage segregation are well characterized in mouse [3, 4]. Although TE initiation is generally conserved among different species including human, cow and mouse [5, 6], studies in human embryos demonstrated fundamental differences in spatial and temporal expressions of TE and ICM determinants between humans and mice [7, 8], highlighting the importance of investigation on human TE specification [9].

Substantial epigenetic reprogramming occurs after fertilization, leading to the formation of a totipotent zygote and subsequent cell fate determination of the embryonic or extra-embryonic tissues [10, 11]. TE determinants accompanied by epigenetic modifications ensure a stable inheritance of cell fate [12] and are crucial for successful embryo implantation and placental development [13]. DNA methylation is one of the critical epigenetic modifications during embryonic development. It involves the covalent addition of methyl group to the fifth position of cytosine (5mC) by DNA methyltransferases (DNMTs) [14] and active oxidization of 5mC into 5-hydroxymethylcytosine (5hmC), 5-formylcytosine (5fC), and 5-carboxylcytosine (5caC) by ten-eleven translocation methylcytosine dioxygenases (TETs) [15]. It is known that loss of DNA methylation can permit changes in cell fate commitment during early embryogenesis. A remarkable reduction of DNA methylation was observed from the 8-cell to blastocyst stage in human embryos, which is accompanied by upregulations of *DNMT3A, DNMT3B*, *TET1* and *TET2* but downregulation of *DNMT1* [16, 17]. Although a global increase in DNA methylation was observed after implantation, TE exhibited a comparatively lower level of DNA methylation as compared to the epiblast [17]. However, the expression patterns of enzymes mediating DNA methylation during TE development and their roles in early trophoblast differentiation are not fully understood. Previous study demonstrated that the loss of DNA methylation prompted trophoblast differentiation from mouse *Dnmt1*-KO embryonic stem cells (ESCs) and DNA demethylation at *Elf5* promoter and induction of its expression reinforced trophoblast fate commitment [18]. Later, researchers discovered 10 genes as the potential epigenetic gatekeepers of the trophoblast identity through screening for hypermethylated promoters between mouse ESCs and trophoblastic stem cells (TSCs) [19]. To date, only hypomethylated *ELF5* has been confirmed in human trophoblast lineages [20]. On the other hand, the initiation of ELF5 during early trophoblast differentiation occurs before its promoter demethylation in humans [21]. It is unclear if the activation of TE-specific genes after loss of DNA methylation is essential for normal human trophoblast development.

The availability of human embryos for scientific research is limited due to ethical concerns. We have established TE-like spheroids from human embryonic stem cells (hESC) [22]. Upon 48 h of differentiation, trophoblastic spheroids at 48 h exhibit a blastocoel-like structure and share similar transcriptomic characteristics with TE of early human blastocysts. Upon further differentiation to polar-TE-like cells, trophoblastic spheroids at 72 h can specifically adhere to the receptive endometrial epithelial cells (EEC) [22, 23] and invade the EEC and endometrial stromal cells [22]. Our clinical trial demonstrated that its attachment rate onto EEC can predict the cumulative livebirth of women aged ≥ 35 [24]. Recently, we have derived expanded potential stem cells from human preimplantation embryos (hEPSC-em) donated for research use. Transcriptomic analysis showed that hEPSC-em resembled morula-stage human blastomeres [25]. When compared to the hESC-derived trophoblastic spheroids, hEPSC-em lines exhibited higher efficiency in trophoblast differentiation with increased expressions of trophoblast markers [25]. Besides, hEPSC-em can differentiate efficiently to TSC [25] as a valuable tool for investigation of early trophoblast development and function [26].

In this study, we aimed to delineate the roles of DNA methylation and to identify TE-specific marker activated by loss of DNA methylation during early trophoblast differentiation. With the use of the morula-like hEPSC-em cells, we demonstrated that inhibition of TETs impeded human trophoblast differentiation. Specifically, we report that glutamyl aminopeptidase (ENPEP) was epigenetically activated by TET2-mediated loss of DNA methylation. Both TET2 and ENPEP played crucial roles in human trophoblast fate commitment.

## Materials and methods

### hEPSC-em culture

The hEPSC-em and hEPSC-em-GATA3^mCherry^ reporter cell lines were cultured and maintained as previously reported [25]. Briefly, the cells were cultured on STO feeder cells mitotically inactivated by mitomycin-C (Thermo Fisher Scientific, USA) or gamma irradiation (Gammacell® 3000 Elan, Nordion, Canada). hEPSC-em culture medium was prepared as follows: Dulbecco’s Modified Eagle’s Medium/Nutrient Mixture F-12 Ham (DMEM/F12), 1% l-glutamine, 1% penicillin–streptomycin, 1% NEAA, 0.1 mM 2-mercaptoethanol, 0.5% N-2 supplement, 1% B-27 supplement (Thermo Fisher Scientific), 65 µg/mL l-ascorbic acid 2-phosphate (Sigma-Aldrich, USA), 2.5 µM XAV939 (Sigma-Aldrich), 0.15 µM A419259 trihydrochloride (Tocris Bioscience, UK), 1.0 µM CHIR 99021 (Stemgent, USA), 0.25 µM SB 590885 (Tocris Bioscience) and 10 ng/mL recombinant human leukemia inhibitory factor (LIF, Peprotech, USA). The medium was changed daily, and the cells were regularly passaged every 3–4 days using 0.05% trypsin. On the day of seeding, 20% Knock-out serum replacement (KOSR, Thermo Fisher Scientific) and 10 μm Y-27632 (STEMCELL Technologies, USA) were supplemented to the hEPSC-em culture medium.

### BAP-induced trophoblast differentiation from hEPSC-em

The hEPSC-em were differentiated into trophoblast cells using a 2-dimensional (2D) or 3D model according to published protocols [22, 27]. The trophoblast differentiation medium consisted of mouse embryonic fibroblast conditioned medium (MEF-CM): Knock-out DMEM, 15% KOSR, 1% NEAA, 1% penicillin–streptomycin, 1% l-Glutamine, and 0.1 mM 2-mercaptoethanol. The BAP supplementation referred to 10 ng/mL BMP4 (R&D system, USA), 1 µM A83-01 (STEMGENT, USA) and 0.1 µM PD 173074 (Sigma-Aldrich). hEPSC-em were digested into single cells with 0.05% trypsin. For 2D differentiation, the cells were seeded at 0.25 × 10^5^ cells/cm^2^ on pre-coated matrigel (Corning) plates in MEF-CM medium supplemented with 10 µM of Y27632. From the second day onwards, the cultured medium was switched to MEF-CM medium containing BAP, and it was changed every day thereafter. For 3D trophoblastic spheroid differentiation, the cells were seeded into AggreWell 400 plate (STEMCELL Technologies) pretreated with Anti-Adherence Rinsing Solution at a density of 150 cells/microwell in MEF-CM medium supplemented with 10 µM of Y27632. The cells were aggregated for 24 h and then transferred into ultra-low adherence 6-well plates (Corning) in MEF-CM medium supplemented with BAP. The medium was changed daily.

### Derivation of TSC lines from hEPSC-em

TSC-em lines were derived from hEPSC-em as described [26] with minor modifications. Briefly, hEPSC-em were digested into single cells and seeded onto geltrex (Corning) coated plate at a density of 0.5 × 10^4^ cells/cm^2^. The cells were passaged after reaching 70%–80% confluency with Tryple Express (Thermo Fisher Scientific). TSC-like colonies appeared after 3–5 passages and stable TSC lines were formed after 8–10 passages. The hTSC culture medium was prepared as described [26]: DMEM/F12 was supplemented with 1% l-glutamine, 0.5% penicillin–streptomycin, 0.3% Bovine serum albumin (BSA) (FUJIFILM Wako, Japan), 0.2% Fetal bovine serum (FBS), 1% ITS-X supplement (Thermo Fisher Scientific), 0.1 mM 2-mercaptoethanol, 50 µg/mL l-ascorbic acid 2-phosphate, 50 ng/mL recombinant human EGF (R&D system), 2 µM CHIR-99021, 0.5 µM A83-01, 1 µM SB 431542, 0.8 mM VPA (FUJIFILM Wako) and 5 µM Y-27632.

STB and EVT differentiations from TSC-em were conducted according to a previous protocol [26] with minor modifications. The basal medium for STB and EVT differentiations was prepared as follows: DMEM/F12, 0.1 mM 2-mercaptoethanol, 0.5% penicillin–streptomycin, 0.3% BSA, 1% ITS-X supplement and 4% KOSR. TSC-em were digested into single cells and seeded at a density of 0.2 × 10^5^ cells/cm^2^ onto matrigel (Corning) coated plates. During the first 3 days of EVT differentiation, the cells were cultured with basal medium supplemented with 7.5 μM A83-01, 100 ng/ml NRG1 (R&D System), 2.5 μM Y27632 and 2% Matrigel (Corning). On day 3, the medium was refreshed using basal medium with 7.5 μM A83-01, 2.5 μM Y27632 and 0.5% Matrigel. For the STB differentiation, cells were cultured in basal medium supplemented with 2.5 μM Y27632 and 2 μM Forskolin (FUJIFILM Wako) and the medium was changed every three days. On day 6, the EVT and STB cells were collected for gene expression analysis.

### Trophoblastic spheroid attachment assay and spreading area calculation

The attachment of hEPSC-em-derived trophoblastic spheroids onto Ishikawa cells, a receptive endometrial epithelial cell derived from human endometrial adenocarcinoma [28] which commonly used for early implantation study [22, 29], was conducted according to the published protocols [22, 23]. The attachment rates were calculated as the number of firmly attached trophoblastic spheroids out of the total number of seeded trophoblastic spheroids. The attached trophoblastic spheroids were co-cultured with Ishikawa cells overnight. The spreading area of trophoblastic spheroids into Ishikawa cell was calculated by the software ImageJ (National Institutes of Health, USA). Briefly, the outline of each spread trophoblastic spheroid was manually drawn [22] and the area was then analysed by the “Measure” function of ImageJ.

### 5-AzaC and DMOG treatment to BAP-induced trophoblast differentiation

5-Azacytidine (5-AzaC, Sigma-Aldrich, USA) stock solution at a concentration of 500 µM in DMEM/F12 supplemented with 10% KOSR was prepared. A 5 mM dimethyloxallyl glycine (DMOG, MCE, USA) stock solution in 11.42 mL Milli Q water was also prepared. Different concentrations of 5-Aza (0.1, 0.2, 0.5, and 1.0 µM) and DMOG (10, 20, 40, and 80 µM) were used for treatments during BAP-induced trophoblast differentiation from our established hEPSC-em-GATA3^mCherry^ reporter cell line [25]. The GATA3^mCherry^ fluorescent signal was detected by Infinite 200 PRO microplate reader (Tecan, Switzerland) at a wavelength of 580 nm.

### Gene knockout in hEPSC-em using CRISPR-Cas9 approach

Two gRNAs targeting exon 3 (3′-TGGAGAAAGACGTAACTTCG-5′) and exon 6 (3′-CGGAGCTTACCGAGACGCTG-5′) of the TET2 locus, and two gRNAs targeting exon 2 (gRNA1: 3′-AAGAATACGGAGCACTTTCA-5′) and exon 10 (gRNA2: 3′-GTGAAAGAAGTAATGGACAC-5′) of the ENPEP locus were inserted into the pKLV2-U6-gRNA-PGK-puro vectors (Addgene #67974) for deletion of the respective genes. Cas9 vector (6 μg of pKLV2-EF1a-Cas9Bsd; Addgene #68343) and 3 μg of gRNA vectors were electroporated into hEPSC-em using a Neon transfection system (Thermo Fisher Scientific) with 1 pulse of 1400 V for 20 ms. The electroporated cells were selected with 10 µg/mL blasticidin and 2 µg/mL puromycin. To confirm the correct deletions, the regions covering the gRNA cutting sites were PCR amplified for Sanger sequencing at the Centre for PanorOmic Sciences (CPOS), the University of Hong Kong. The Sanger sequencing results were visualized by the SnapGene Viewer (Dotmatics, https://www.snapgene.com/). Deletion of ENPEP and TET2 proteins were validated using Western Blotting or immunocytochemistry. The PCR primers and antibodies used are listed in Supplementary Tables S1 and S2, respectively.

### Bisulfite sequencing

Genomic DNA was bisulfite converted using the EpiTect Plus DNA Bisulfite Kit (QIAGEN, German) following manufacturer’s instruction. The converted DNA was subjected to PCR amplification using the Meth-Primer of ENPEP designed by an online platform [30]. The CpG islands in the promoter of ENPEP were identified. The primer sequences used were: Forward primer (F1): TTTAGGTTGAGTGGTAAAGGTTGAG; Reverse primer (R1): TACAAAAAAATTATCACAACTCCCC. The PCR products were purified using the GeneJET PCR Purification Kit (Thermo Fisher Scientific) and ligated into the pGEM®-T Easy vector (Promega). Blue/White colony screening for successful integration was conducted using the X-Gal/IPGT LB-agar plates. The white bacteria colonies were picked and expanded in LB-Broth. Plasmid DNA was extracted using the QIAprep® Spin Miniprep Kit (QIAGEN, German). The DNA was Sanger sequenced using the T7 primer (5′ TAATACGACTCACTATAGGG 3′) and SP6 primer (5′ ATTTAGGTGACACTATAG 3′) by CPOS. The unmethylated CpG sites (CG) in the original DNA were converted into UG after sodium bisulfite treatment and the methylated CpG sites (mCG) were unmodified. The proportion of methylated cytosines was defined as the number of methylated CpG sites out of the total CpG sites.

### Real-time quantitative polymerase chain reaction (RT-qPCR)

Total RNA was isolated using the mirVana PARIS RNA kit (Thermo Fisher Scientific) and was reverted to complementary DNA (cDNA) by the PrimeScript Reverse Transcription Kit (Takara, Japan). RT-qPCR was performed using the TaqMan Gene Expression Assay in an Applied Biosystems QuantStudio™ 5 Real-Time PCR System (Thermo Fisher Scientific). The mRNA levels were quantified using the 2^−ΔΔCT^ method and normalized with the endogenous 18S ribosomal RNA. The probes used are listed in Supplementary Table S3.

### Chromatin immunoprecipitation (ChIP)-qPCR

hEPSC-em, trophoblastic spheroids at 24 h and trophoblastic spheroids at 48 h were collected for ChIP using the SimpleChIP Enzymatic Chromatin IP Kit (Cell Signaling Technology, USA). To digest the nucleus DNA to a length of 150–900 bp, 0.5 µL of Micrococcal Nuclease followed by sonication (Soniprep 150, MSE) was adopted. For each IP sample, 5 µg of digested, cross-linked chromatin was incubated with 2 µg of antibody overnight. Normal rabbit IgG was set as negative control and the rabbit anti-Histone H3 antibody was used as positive control. The DNA fragments bound with target proteins were pulled down by the Protein G Magnetic Beads. After cross-linking reversal and DNA purification, the DNA was subjected to RT-qPCR. The DNA level relative to the Input was calculated using the formula: 2% × 2^(Ct value of 2%Input Sample – Ct value of IP Sample)^. The antibodies and primers used are listed in Supplementary Tables S2 and S4, respectively.

### Immunofluorescence staining

Cells were fixed in 4% paraformaldehyde and permeabilized with 0.1% Triton X-100 (Sigma-Aldrich). After blocking with 10% normal goat serum, the cells were incubated with primary antibodies at 4 °C overnight. In the next day, the cells were incubated with fluorescence-conjugated secondary antibodies (Thermo Fisher Scientific). The nuclei were stained with Hoechst 33258 (Thermo Fisher Scientific). Images were captured using the confocal microscope (LSM 980, Carl Zeiss AG) at the CPOS. The antibodies used in this study are listed in Supplementary Table S2.

### Detection of hCG in conditioned medium

Spent culture media were collected and the hCG level in the media was detected by the ARCHITECT total β-hCG reagent kit (Abbott, USA) according to the manufacturer’s instruction.

### Cell viability assay (XTT)

The XTT assay was performed using the Cell Proliferation Kit II (Sigma-Aldrich) according to the manufacturer’s instructions. Briefly, the culture medium was replaced with phenol red free DMEM/F12 medium and XTT reagents were then added to each well. After incubation at 37 ℃ for 4 h, the absorbance was detected by an Infinite 200 PRO microplate reader at a wavelength of 450 nm.

### Bioinformatics analysis

The published RNA sequencing datasets used in this study included pre-implantation [31] and post-implantation (GSE136447) [32] human embryos profiled by single-cell RNA sequencing. The violin plots were plotted with the R package ggplot2 using log_2_(TPM) or log_2_(count) values calculated for target genes across different samples. The published reduced representation bisulphite sequencing (RRBS) datasets analyzed in this study were obtained from GSE49828 [16] and analyzed with the R package MethylKit [33]. The hypo- and hyper-methylated CpGs were defined as methylation differences greater than 25% with *p* < 0.01. Promoters were defined as regions 1 kb upstream and downstream of transcriptional start sites (TSS). The differentially methylated CpGs within the promoter region were then mapped to the human hg38 genome using the R package genomation and annotated in Ensembl. The Ensembl ID was then converted to Gene symbols using the R package biomaRt. An online platform (https://bioinformatics.psb.ugent.be/webtools/Venn/) was used to generate the Venn diagram.

### Statistics

Data were analyzed and plotted using Prism 9 (Graphpad). The normal distribution of data was assessed using normality test of Shapiro–Wilk (W) and Kolmogorov–Smirnov (Distance). The *p* values were calculated using unpaired t-test if data were normally distributed. Otherwise, Mann–Whitney U test was performed. The threshold for statistical significantly difference was *p* < 0.05.

## Results

### Differential expression patterns of enzymes mediating DNA methylation during early trophoblast development

**Fig. 1:**
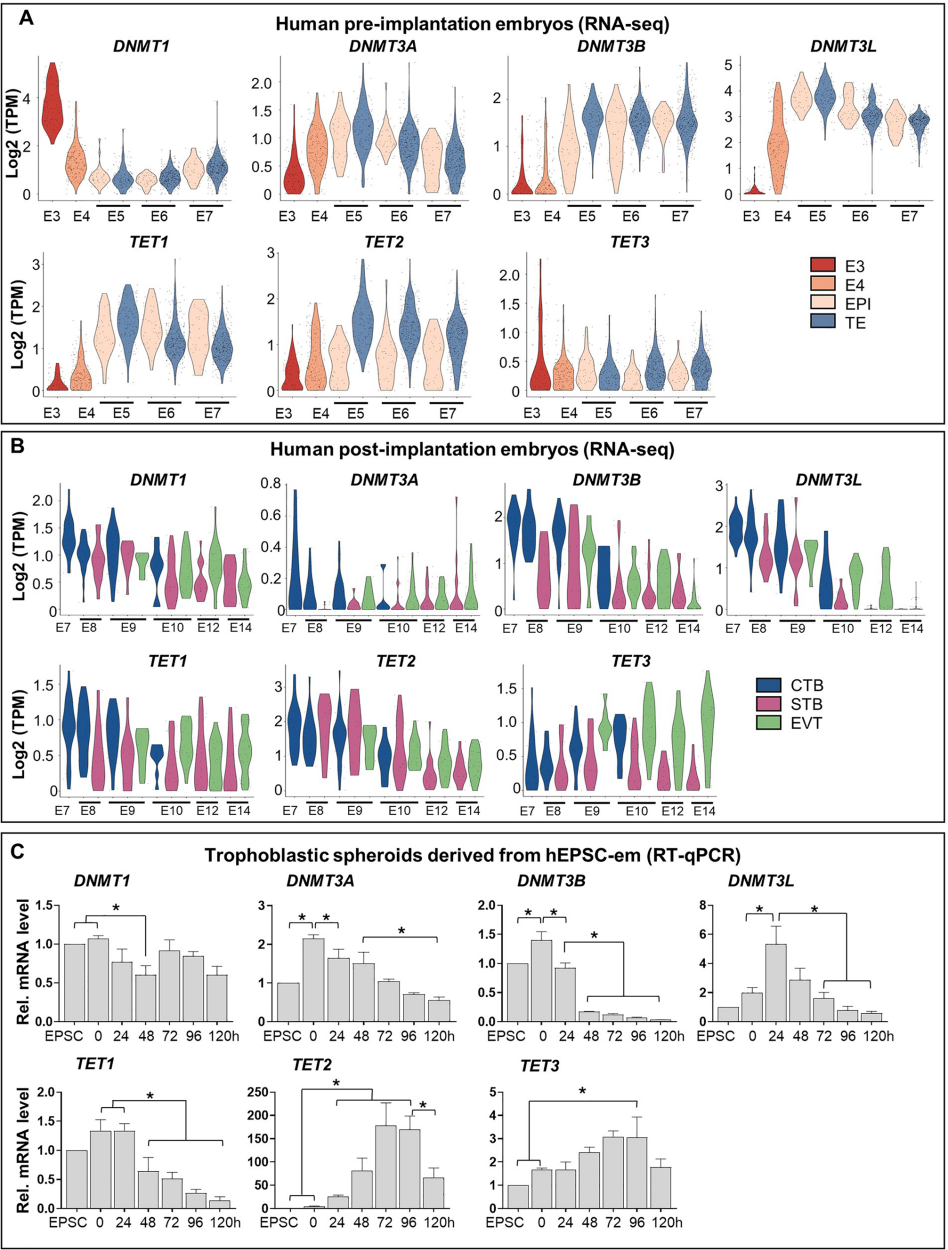
Expression patterns of DNMTs and TETs in early human embryos and hEPSC-em derived trophoblastic spheroids. **A**,** B** The gene expression patterns of *DNMTs* (*DNMT1*, *DNMT3A*, *DNMT3B* and *DNMT3L*) and *TETs* (*TET1*, *TET2* and *TET3*) in TE and EPI of human preimplantation embryos from embryonic day 3 (E3) to E7 (**A**) and human post-implantation embryos from E7 to E14 (**B**). **C** The relative mRNA levels of *DNMTs* and *TETs* during trophoblastic spheroid differentiation from hEPSC-em at 0 h to 120 h. Data were presented as mean ± SEM, n = 3. *t-test* was performed. *p < 0.05

The differential expression patterns of DNMTs and TETs were analysed using published datasets of pre- and post-implantation embryos [31, 32]. It was found that *DNMT3A*, *DNMT3B*, *DNMT3L*, *TET1* and *TET2* were induced substantially from embryonic day 3 (E3) to day 5 (E5). While *DNMT1* was decreased substantially, constant expression of *TET3* was observed within the same period (Fig. 1A). Notably, *TET2* was the only gene showing higher expression level in TE than in EPI from E5 to E7 (Fig. 1A). We further analysed the dataset of post-implantation embryos from E7 to E14 [32], downregulations of *DNMTs* and *TETs* were observed during differentiation of CTB into STB and EVT (Fig. 1B). The mRNA expression levels of these two family members were studied in our trophoblastic spheroid differentiation model, in which early TE-like and trophoblast-like signatures were induced upon induced differentiation for 48 h and 96 h, respectively [23, 25]. As expected, quantitative PCR analysis demonstrated similar expression patterns of these enzymes during trophoblastic differentiation from hEPSC-em (Fig. 1C); the initially induced *DNMT3* members and *TET1* were gradually down-regulated upon further trophoblast differentiation. Similarly, down-regulation of *DNMT1* was observed at the first 48 h of differentiation. Although both *TET2* and *TET3* expressions were induced from 0 to 96 h, significant induction of *TET2* occurred from 24 h post-differentiation whereas significant augmentation of *TET3* was only observed at 96 h (Fig. 1C). These results suggested that the downregulation of DNMTs and upregulation of TETs might play critical roles in early trophoblast development.

### Effects of DNMTs and TETs inhibitors on trophoblast differentiation

**Fig. 2:**
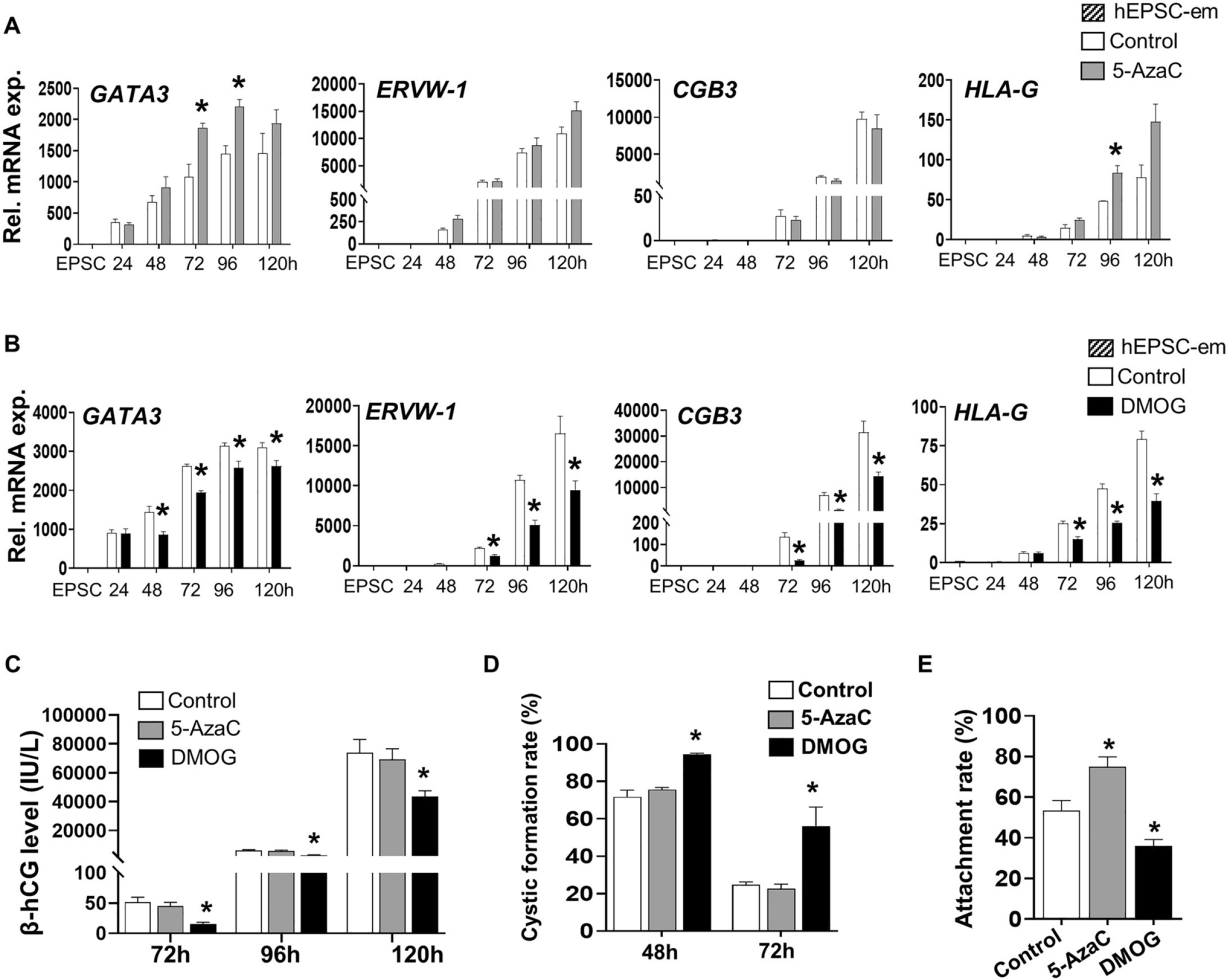
Effects of 5-AzaC and DMOG on trophoblast differentiation and trophoblastic spheroid formation. **A**, **B** The relative mRNA expression of early trophoblast marker *GATA3*, STB markers (*ERVW-1* and *CGB3*) and EVT marker (*HLA-G*) after treatments with 0.5 μM of 5-AzaC (0–24 h) (**A**) or 40 μM of DMOG (24-48 h) (**B**) during BAP-induced trophoblast differentiation from hEPSC-em-GATA3^mCherry^ reporter line (n = 3). **C** The β-hCG levels in the conditioned media collected from 72 to 120 h after treatments with 0.5 μM of 5-AzaC at 0-24 h or 40 μM of DMOG at 24-48 h during BAP-induced trophoblast differentiation from hEPSC-em-GATA3^mCherry^ reporter line **(**n = 3).** D** The percentages of cystic structure formed in control, 5-AzaC or DMOG treated trophoblastic spheroids at 48 h and 72 h (n = 4–5). **E** The 1-h attachment rates of control, 5-AzaC- and DMOG-treated trophoblastic spheroids at 72 h on receptive Ishikawa cells (n = 4). Data were presented as mean ± SEM. *p < 0.05 compared to control group

We utilized 5-AzaC (DNMTs inhibitor) [34] and DMOG (TETs inhibitor) [35] that altered global DNA methylome for studying the effects of methylation on trophoblast differentiation. The time- and dose-dependent effects of both chemicals on BAP-induced trophoblast differentiation were first investigated using our hEPSC-em-GATA3^mCherry^ reporter cell line [25]. The treatments only covered the first 48 h of trophoblastic spheroid formation when differentiation of the TE lineage was initiated [23]. The different doses of 5-AzaC (0.1, 0.2, 0.5, and 1.0 μM) and DMOG (10, 20, 40, and 80 μM) used did not affect cell viability of the differentiating cells (Fig. S1A) within a period of 120 h. While treatments with 0.5 μM and 1.0 μM of 5-AzaC at 0–24 h significantly increased the GATA3^mCherry^ signal at 120 h, treatments with 40 μM and 80 μM of DMOG at 24–48 h significantly reduced the GATA3^mCherry^ signal (Fig. S1B). We further analysed the gene expressions of different trophoblast markers in the 0.5 μM 5-AzaC (0–24 h) and 40 μM DMOG (24-48 h) treated cells. While 5-AzaC had no effect on the expressions of pluripotent marker *OCT4*, it significantly increased the expressions of markers of early trophoblast (*KRT7* and *GATA3*) and EVT (*HLA-G*) (Fig. 2A, Fig. S1C). In contrast, treatment of DMOG significantly decreased the expressions of *GATA3*, *ERVW-1*, *CGB3* and *HLA-G* at 72–120 h after BAP treatment and delayed the reductions of *KRT7* at 96 h and 120 h (Figs. 2B, S1D), when the early trophoblast marker *KRT7* was reported to decrease at these two time points [25]. Concordantly, DMOG significantly lowered the levels of β-hCG secretions while no significant effect was observed in the 5-AzaC treated group (Fig. 2C). The effects of 5-AzaC and DMOG on trophoblastic spheroid formation and attachment onto receptive EEC (Ishikawa cells) were investigated using our published protocol [22]. Upon 48 h of trophoblast differentiation, around 70% of trophoblastic spheroids at 48 h formed cystic structures in the control and the 5-AzaC treated groups, and most of the cystic structures collapsed at 72 h post-differentiation. However, treatment with DMOG significantly delayed the collapse of the cystic structures, leading to significantly higher cystic formation rates at 48 h and 72 h (Figs. 2D, S1E). The attachment rate of 5-AzaC treated trophoblastic spheroids at 72 h was significantly higher than the control group, while DMOG significantly reduced the attachment rate of trophoblastic spheroids onto the Ishikawa cells (Fig. 2E). These results suggested that loss of DNA methylation is a critical process for proper trophoblast differentiation.

### ENPEP is a TE marker regulated by DNA methylation

**Fig. 3:**
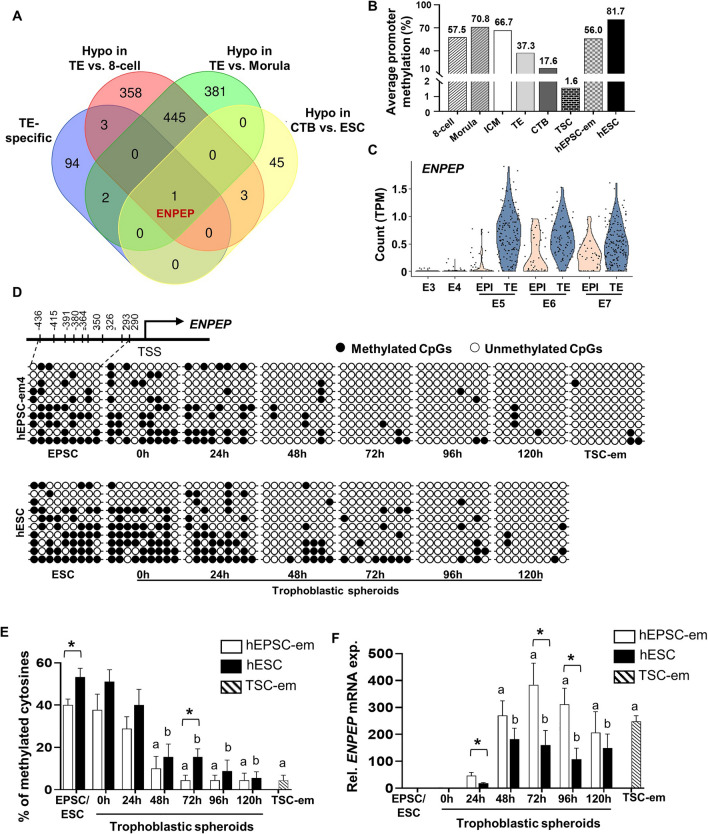
Identification of ENPEP as a candidate TE-marker regulated by DNA methylation. **A**)Venn diagram showing the clustering of hypomethylated genes in TE (verse 8-cell and morula) and CTB (verse hESC) with the top 100 TE-specific genes. **B** The average DNA methylation levels of *ENPEP* promoter in pre-implantation embryos (8-cell embryos, morula stage embryos, ICM, TE) [16], CTB [26], TSC derived from CTB [26] and hESC [26] obtained from published datasets and hEPSC-em (this study). **C** Violin plot showing the expression patterns of *ENPEP* in published dataset of human preimplantation embryos [31]. **D**, **E** Representative bisulfite sequencing results (**D**) and the average percentages of methylated cytosines (**E**) of *ENPEP* promoter in hEPSC-em- and hESC-derived trophoblastic spheroids at different time points, and in TSC-em. Each horizontal line represented the sequencing result of one clone. Filled circles and open circles represented the methylated and unmethylated CpG dinucleotide, respectively. **F** The relative mRNA expression of *ENPEP* in hEPSC-em- and hESC-derived trophoblastic spheroids and in TSC-em was determined by RT-qPCR. Data were presented as mean ± SEM, n = 4. *t-test* was performed for gene expression analysis. Mann–Whitney U test was performed for DNA methylation analysis. ^a^*p* < 0.05 when compared to hEPSC-em; ^b^*p* < 0.05 when compared to hESC; *p < 0.05 pairwise comparison between trophoblastic spheroids derived from hEPSC-em and hESC at the same time points

To identify TE determinants that were regulated by DNA methylation, we compared four sets of genes in the published datasets: hypomethylated genes in TE relative to 8-cell embryos and to morulae [16], hypomethylated genes in CTB relative to hESC [26], and the top 100 TE specific genes in preimplantation embryos [31]. It was found that glutamyl aminopeptidase (ENPEP), a trophoblast progenitor marker [36], was commonly found in these 4 gene sets (Fig. 3A). The average DNA methylation levels in the promoter of *ENPEP* in different cell types in the published datasets [16, 26] were analyzed. The *ENPEP* promoter displayed high levels of DNA methylation at 8-cell (57.5%), morula (70.8%) and ICM of blastocyst (66.7%) stages. Its level in TE (37.3%) was comparably lower (Fig. 3B). Much lower methylation levels were observed in CTB (17.6%) and TSC-derived CTB (1.6%) when compared to hESC (81.7%) and hEPSC-em (56.0%) (Fig. 3B). Concomitantly, analysis on single-cell RNA sequencing data of preimplantation embryos [31] demonstrated a profound induction of *ENPEP* at E5 TE as compared to embryos at E3 (8-cell) and E4 (morula), and its levels were manifestly higher in TE when compared to EPI from E5 to E7 (Fig. 3C).

Using the online platform MethPrimer, one CpG island was identified in the *ENPEP* promoter (Fig. S2). Bisulfite sequencing was used to investigate the differential DNA methylation levels in the *ENPEP* promoter during trophoblast differentiation. The promoters were differentially methylated between hEPSC-em and hESC, with average percentages of methylated cytosine of 40% ± 2.89% and 53.33% ± 4.08%, respectively (Fig. 3D and E). In hEPSC-em- and hESC-derived trophoblastic spheroids, the DNA methylation levels of the *ENPEP* promoter decreased significantly at 48 h and maintained at low levels thereafter (Fig. 3D and E). Concordantly, pronounced inductions of *ENPEP* mRNA expression was observed from 48 h onwards (Fig. 3F). Notably, hEPSC-em derived trophoblastic spheroids exhibited a faster loss of DNA methylation in the *ENPEP* promoter and higher gene expression when compared with the hESC-derived trophoblastic spheroids, reflecting better trophoblast differentiation potency of hEPSC-em as reported [25]. Moreover, *ENPEP* was hypomethylated and highly expressed in TSC-em (Fig. 3D–F). Immunofluorescence staining confirmed the protein expressions of ENPEP in hEPSC-em-derived trophoblastic spheroids and TSC-em (Fig. S3).

### ENPEP is essential in trophoblast cell fate commitment

**Fig. 4:**
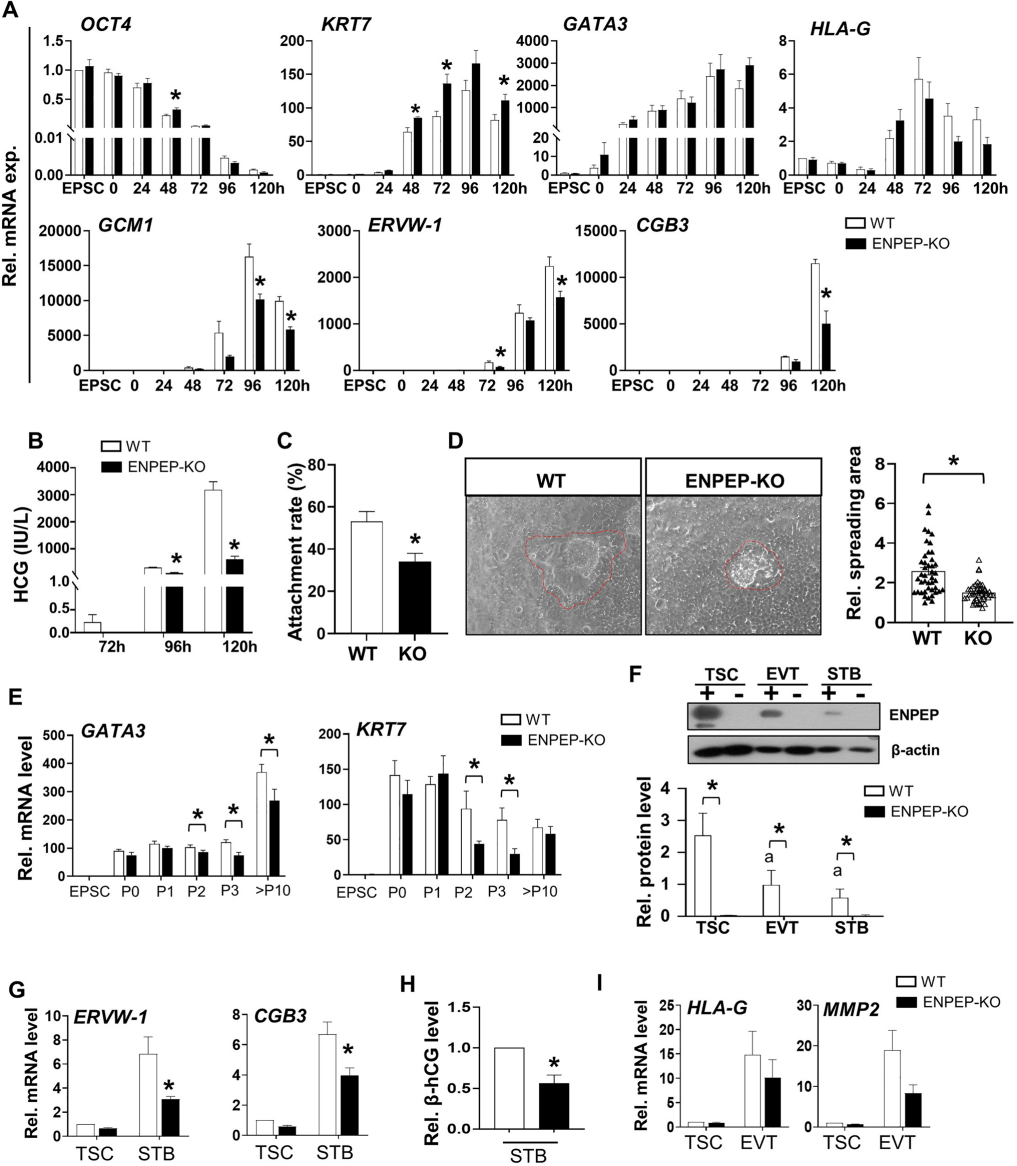
Deletion of ENPEP impaired trophoblast differentiation from hEPSC-em. **A** The relative mRNA expression of pluripotent marker (*OCT4*), early trophoblast markers (*KRT7* and *GATA3*), EVT marker (*HLA-G*) and STB markers (*GCM1*, *ERVW-1* and *CGB3*) as determined by RT-qPCR in trophoblastic spheroids generated from WT and *ENPEP*-KO hEPSC-em. (n = 3–4). **B** The β-hCG secretion levels in the conditioned media of trophoblastic spheroids from 72 to 120 h (n = 3). **C** The attachment rates of WT and *ENPEP*-KO hEPSC-em derived trophoblastic spheroids at 72 h after 1 h of coculture with receptive Ishikawa cells (n = 5). **D** Representative pictures of trophoblastic spheroids cocultured with Ishikawa cells for 24 h (left) and the spreading area of trophoblastic spheroids. **E** The relative mRNA expression of TSC markers (*GATA3* and *KRT7*) during TSC derivation from WT and KO hEPSC-em at passage 0–3 and passage 10–12 (n = 4).** F** Western blotting analysis of ENPEP protein levels in TSC, EVT and STB differentiated from TSC (n = 4). **G**, **H** The relative mRNA expressions of STB markers (*ERVW-1* and *CGB3*) as determined by RT-qPCR (n = 4–5) (**G**) and the β-hCG secretion levels in the spent culture media of differentiated STB from WT and KO TSC-em as detected by enzyme immunoassay (n = 6) (**H**). **I** The relative mRNA expressions of EVT markers (*HLA-G* and *MMP2*). Data were presented as mean ± SEM. *t-test* was performed for gene and protein expression analysis. Mann–Whitney U test was used for the analysis of attachment rates and β-hCG levels. ^a^*p* < 0.05 when compared to WT hEPSC-em or WT TSC-em; *p < 0.05 pairwise comparison between WT and *ENPEP*-KO group at the same time points

ENPEP is a trophoblast progenitor marker [36], but its role during trophoblast differentiation is unknown. We knockout *ENPEP* (*ENPEP*-KO) in the hEPSC-em line using the CRISPR-Cas9 approach (Fig. S4A). The deletions of ENPEP in hEPSC-em and its derived trophoblasts were confirmed by Sanger sequencing (Fig. S4B), quantitative PCR (Fig. S4C) and western blotting analyses (Fig. S4D). The gene expressions during trophoblast differentiation were compared between the *ENPEP* wildtype (WT) and *ENPEP*-KO hEPSC-em lines. The results showed that *ENPEP* deletion delayed the downregulation of *OCT4* at 48 h and early trophoblast marker *KRT7* at 120 h in trophoblastic spheroids, reduced the expressions of STB markers (*GCM1*, *ERVW-1*, and *CGB3*) but had no effect on the expression of EVT marker *HLA-G* (Fig. 4A). In agreement, β-hCG secretion was significantly lower in the *ENPEP*-KO trophoblastic spheroids (Fig. 4B). Moreover, the attachment rates of *ENPEP*-KO trophoblastic spheroids onto EEC were significantly lower than the WT group (Fig. 4C). After co-cultured with EEC for 24 h, the average spreading area of trophoblastic spheroids was significantly smaller in the *ENPEP*-KO group (Fig. 4D).

TSC lines were derived from the WT and the *ENPEP*-KO hEPSC-em lines. Downregulation of *OCT4* and upregulation of TSC markers (*GATA3*, *KRT7* and *TFAP2C*) were observed during TSC derivation (Figs. 4E, S4E). The expression levels of TSC markers were significantly lower in the *ENPEP*-KO group than the WT group in the first 3 passages of TSC derivation but only the *GATA3* gene expression was significantly lower in the *ENPEP*-KO-derived TSC at passages > 10 (Fig. 4E). In addition, TSC devoid of *ENPEP* exhibited lower levels of TSC-specific *miR-525-3p* (Fig. S4F), though HLA-A, -B and -C were undetectable in both the WT and *ENPEP*-KO TSC (Fig. S4G).

The two groups of TSC were induced to differentiate into EVT and STB as reported [25]. Upon inductions of differentiations into EVT and STB, the protein levels of ENPEP were significantly downregulated in the ENPEP-WT cells (Fig. 4F), consistent with the patterns observed in human post-implantation embryos [32] (Fig. S4H). The absence of *ENPEP* in TSC impeded the differentiation as demonstrated by significant reduction of expressions of STB markers (*ERVW-1* and *CGB3*) (Fig. 4G) and β-hCG secretion level (Fig. 4H). Although not statistically significant, decreasing trends of EVT markers (*HLA-G* and *MMP2*) were observed in *ENPEP*-KO group (Fig. 4I). Taken together, deletion of *ENPEP* impaired early trophoblast development, in particular the STB differentiation.

### TET2 mediated loss of DNA methylation and induction of *ENPEP* is essential for early trophoblast differentiation

**Fig. 5:**
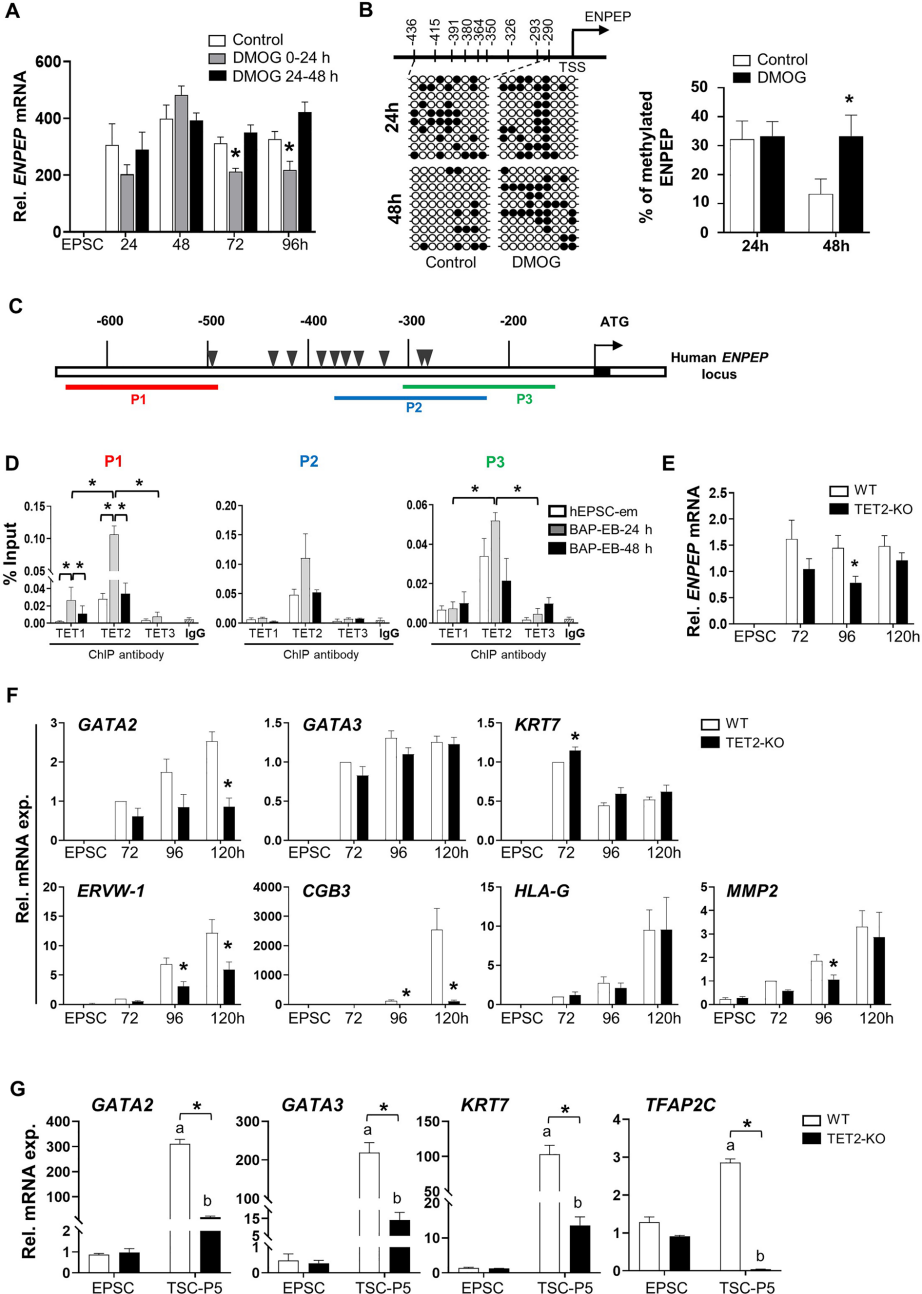
TET2 mediated ENPEP expression and its role during trophoblast differentiation. **A** The relative mRNA expression of *ENPEP* after treatments with 40 μM DMOG at different time points during BAP-induced differentiation (n = 3). **B** Representative bisulfite sequencing results (left) and the average percentages of methylated cytosines (right) of *ENPEP* promoter in untreated (control) and DMOG treated groups at 24 h and 48 h during BAP-induced trophoblast differentiation. Each horizontal line representing the sequencing result of one clone. Filled circles and open circles represented the methylated and unmethylated CpG dinucleotide, respectively. **C** Schematic diagram showing the positions of the primer pairs (P1, P2 and P3) of human *ENPEP* promoter used for ChIP-qPCR analysis. CpG sites were denoted as triangles.** D** ChIP-qPCR results showing the relative enrichment of *ENPEP* promoter DNA by antibodies against TET1, TET2 and TET3 proteins. IgG of the same species were included as the controls for ChIP analysis (lower panel) (n = 3). **E** The relative mRNA expression of *ENPEP* in *TET2*-WT and *TET2*-KO hEPSC-em derived trophoblasts (n = 4). **F** The relative mRNA expression of early trophoblast markers (*GATA2*, *GATA3* and *KRT7*), STB markers (*ERVW-1* and *CGB3*) and EVT marker (*HLA-G* and *MMP2*) during BAP-induced trophoblast differentiation from WT and *TET2*-KO hEPSC-em lines (n = 5). **G** The relative mRNA expression of TSC markers (*GATA2*, *GATA3*, *KRT7* and *TFAP2C*) at passage 5 during TSC derivation from WT and *TET2*-KO hEPSC-em. The data were presented as mean ± SEM. Mann–Whitney U test was performed for DNA methylation analysis. *t-test* was performed for gene expression analysis. ^a^*p* < 0.05 when compared to WT hEPSC-em; ^b^*p* < 0.05 when compared to *TET2*-KO hEPSC-em; *p < 0.05 compared between WT and *TET2*-KO

The involvements of TETs in regulating the expression and methylation of *ENPEP* were investigated by DMOG treatments during trophoblastic spheroid differentiation. Treatment with DMOG at 0-24 h suppressed *ENPEP* expressions at 72 h and 96 h (Fig. 5A) and hindered loss of DNA methylation in *ENPEP* promoter as compared to the control group at 48 h of differentiation (Fig. 5B), suggesting a role of TET proteins in regulating DNA methylation of *ENPEP* promoter. To verify the TET-mediated *ENPEP* expression, enrichment of TET proteins in *ENPEP* promoter was determined by ChIP-qPCR. Three pairs of primers were designed to cover the *ENPEP* promoter regions (P1, P2 and P3) near the CpG sites (Schematic Fig. 5C). The results showed high enrichment of TET2 binding but not that of TET1 and TET3 in all the promoter regions studied (Fig. 5D). Notably, the enrichments of TET2 protein at P1 and P3 were significantly higher than that of TET1 and TET3 in trophoblastic spheroids at 24 h (Fig. 5D). In addition, the binding of TET1 or TET2 at P1 was significantly higher in the trophoblastic spheroids at 24 h than undifferentiated hEPSC-em and trophoblastic spheroids at 48 h, though the enrichment was substantially higher for TET2 (Fig. 5D). Although not statistically significant, increased trends of TET2 binding at P2 and P3 of the *ENPEP* promoter were also observed in the trophoblastic spheroids at 24 h (Fig. 5D). These results suggested that loss of DNA methylation of *ENPEP* involved TET proteins, particularly TET2 during trophoblast differentiation.

We next delineate the role of TET2 during early trophoblast differentiation by knocking out TET2 in hEPSC-em using the CRISPR-Cas9 approach (Fig. S5A). The absence of TET2 in hEPSC-em and its derived trophoblasts was confirmed by Sanger sequencing (Fig. S5B), quantitative PCR (Fig. S5C) and immunofluorescence staining (Fig. S5D). The gene expressions during BAP-induced trophoblast differentiation were measured in wildtype (WT) and TET knockout (*TET2*-KO) hEPSC-em lines. The results showed that *TET2*-KO significantly suppressed the induction of *ENPEP* at 96 h post-BAP treatment, and induced decreased trends of *ENPEP* expression at 72 h and 120 h (Fig. 5E). Meanwhile, *TET2*-KO led to pronounced repressions of STB markers (*ERVW-1* and *CGB3*) but not EVT marker (*HLA-G*) (Fig. 5F). We also attempted to derive TSC from the WT and *TET2*-KO hEPSC-em lines. TSC colonies were established from the *TET2*-KO hEPSC-em at passage 5, with remarkable induction of TSC markers *GATA2*, *GATA3*, *KRT7* and *TFAP2C* (Fig. 5G). However, no TSC-like colonies were observed in the *TET2*-KO group at passage 5 and the expression levels of TSC markers in the remaining cells were significantly lower than those in the WT TSC colonies (Fig. 5G). These results indicated the critical role of TET2 during early trophoblast differentiation.

## Discussion

**Fig. 6:**
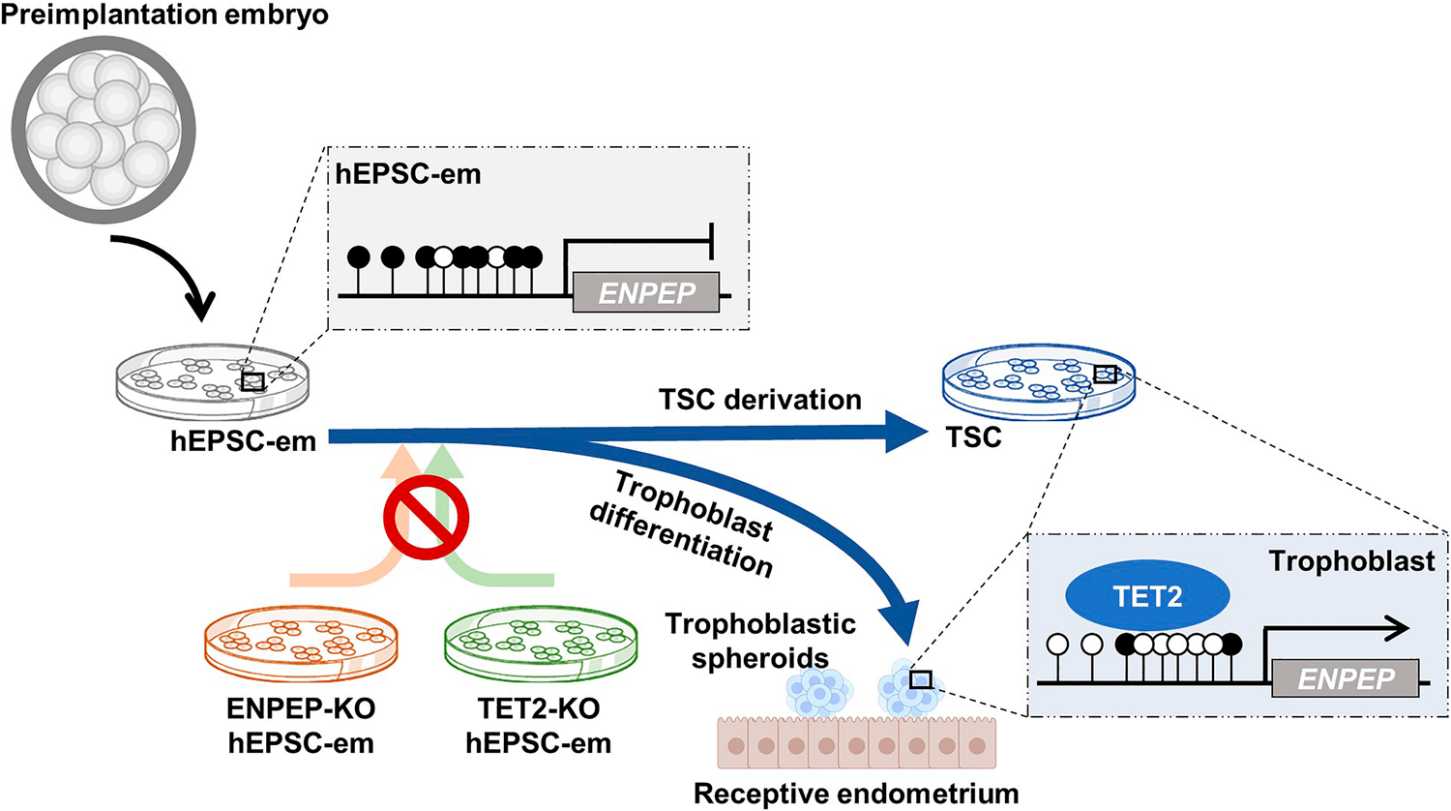
Schematic diagram of TET2-mediated loss of DNA methylation in ENPEP promoter and gene activation during early trophoblast differentiation. Hypermethylated ENPEP in morula-like hEPSC-em is demethylated through TET2 recruitment on ENPEP promoter during trophoblastic spheroid differentiation and TSC derivation. Deletion of ENPEP or TET2 in hEPSC-em compromises trophoblast differentiation potency and reduces adhesion and invasion of derived trophoblastic spheroids

Understanding the intricate processes and deciphering the mechanisms of the first cell differentiation into distinct cell lineages are hampered by limited availability of human embryos. Here, with the use of hEPSC-em with enhanced trophoblast differentiation potential, we demonstrated that TET2-mediated *ENPEP* hypomethylation and gene expression were critical for early trophoblast differentiation (Fig. 6).

The comparable expression patterns of *DNMTs* and *TETs* in hEPSC-em derived trophoblastic spheroids and TSC with the published transcriptomic datasets of human preimplantation [31] and post-implantation [32] embryos supported the use of the current model for the study. As speculated in a previous study [16], the reduced DNA methylation maintainer *DNMT1* and increased DNA methylation erasers *TET1* and *TET2* might contribute to the erasure of parental epigenomes, whereas the upregulations of *DNMT3s* during first lineage specification might be involved in gaining de novo methylation marks in the embryos. The genome-wide DNA methylation profiles of the mid-gestation mouse embryos and placenta revealed that the majority (96.8%) of the differentially methylated regions (DMRs) are hypomethylated in the placenta, resulting in an overall low level of methylation in extraembryonic tissues [37]. Intriguingly, a higher level of *TET2* was observed in TE than the EPI of E5 to E7 human blastocyst. Previous study demonstrated that DMRs are hypermethylated in mouse ESCs devoid of Tet2 when compared to both WT and Tet1-KO ESCs, which are accompanied by a larger number of downregulated genes during differentiation induced by leukemia inhibitory factor (LIF) withdrawal [38]. The significance of TET2 in trophoblast differentiation was further supported by the substantial upregulation of *TET2* expressions in early TE-like trophoblastic spheroids at 48 h and pTE-like trophoblastic spheroids at 72 h.

We then utilized two inhibitors targeting the DNMTs and TETs to first confirm the roles of DNA methylation. The metabolites of 5-AzaC can be incorporated into DNA, forming covalent adducts with cellular DNMTs. This led to the degradation of the covalently trapped enzymes and subsequent rapid passive loss of methylation during DNA replication [34, 39]. On the other hand, DMOG inhibits TET enzyme activities by acting as a small-molecule inhibitor of 2OG-dependent oxygenases [35]. In this study, stimulatory effects of 5-AzaC but inhibitory effects of DMOG were observed during initiation of trophoblast differentiation. Indeed, *Dnmt1* knockout induces efficient trophoblast differentiation in post-implantation epiblast-like mouse ESCs [40] originally lacking trophoblast differentiation potential [41]. On the contrary, triple knockout of Tet1-3 in mouse embryos induced hypermethylation and diminished expressions of *Lefty1* and *Lefty2*, thereby hyperactivating Nodal signaling [42], a critical pathway required to be inactivated during trophoblast differentiation [22]. The differential time-dependent effects of 5-AzaC and DMOG on the expression of trophoblast markers might be attributed to the dissimilar expression patterns of *DNMTs* and *TETs* during early trophoblast differentiation. In particular, the positive effects of 5-AzaC on trophoblast differentiation were exclusively observed when 5-AzaC treatment covered the first 24 h of differentiation during which *DNMTs* were expressed at higher levels. In contrast, obvious effects of DMOG were only observed when the treatment covered 24–48 h of differentiation, which aligned with the gradual induction of *TET2* from 24 to 72 h. The observation might further imply that DMOG induced TET2 inhibition played a critical role in early trophoblast differentiation.

We studied the endometrial attachment competencies of 5-AzaC- or DMOG-treated trophoblastic spheroids. Trophoblastic spheroids at 72 h with collapsed cystic structures secret HCG and are developmentally competent for attaching onto receptive endometrial epithelial cells [22–24]. HCG produced by human embryos can increase the expression of LIF, a marker of endometrial receptivity, thereby enhancing the receptivity of EECs [43]. In this connection, the reduced HCG productions, delayed collapse of cystic structures and reduced attachment rates further reinforced that DMOG treatment reduced the differentiation potential of trophoblastic spheroids into pTE-like stage. Although 5-AzaC had no effect on *CGB3* expression and HCG secretion, it significantly enhanced the attachment rate of trophoblastic spheroids at 72 h. It was reported that 5′-aza-2’-deoxycytidine treatment induced the expressions of E-cadherin and γ-catenin, two adhesion molecules critical for embryo attachment [44, 45], in a trophoblast cell line (BeWo) [46]. Thus, 5-AzaC might also be involved in increasing the expressions of adhesion molecules on the trophoblastic spheroids, leading to higher EEC attachment potential. Taken together, the opposite effects of 5-AzaC and DMOG highlighted the crucial roles of DNA methylation during trophoblast differentiation.

Recent innovative techniques such as low-input chromatin analysis have emerged to broaden our accessibility of human early-stage embryos and discover the mechanisms of DNA methylation modifications on a whole-genome-wide level [16, 47, 48]. With the substantial inductions of TETs and higher impacts of DMOG during early trophoblast differentiation, we identified epigenetically regulated genes in human TE. By matching the published DNA methylomes of human embryos [16, 26] with TE-specific genes [31], ENPEP was identified as a hypomethylated gene in early TE lineages when compared to 8-cell/morula stage human embryos and epiblast-like hESC. Further validations confirmed the gradual loss of DNA methylation and induction of *ENPEP* expression during the first 72 h of trophoblastic spheroid differentiation. An observed decline of *ENPEP* gene expression at later time points when DNA methylation remained low could be due to other regulatory mechanisms on *ENPEP* during trophoblast subtypes establishment. For examples, the 3′UTR region of *ENPEP* contained several miRNA binding sites [49], which may contribute to the reductions of *ENPEP* at later time points when its DNA methylation level remained low. Similar to the reported findings that more rapid loss of DNA methylation in the *ELF5* promoter and its higher expression in the hEPSC-em derived trophoblastic spheroids as compared to that in the primed hESC [25], the faster demethylation and higher expression of *ENPEP* in hEPSC-em derived trophoblastic spheroids might contribute to the expanded potential of hEPSC-em into trophoblast lineages.

ENPEP is a cell surface marker of TE progenitor cells [21, 36, 50], but its roles in trophoblast development is unknown. In human placenta, ENPEP was predominantly expressed in the apical membrane of CTB and on the surface of apical microvilli of STB to act as a component of the fetal-maternal barrier to oligopeptides [51]. Here, we demonstrated that the deletion of *ENPEP* impaired trophoblast differentiation. Placental development initiates in a hypoxic environment, where the induced hypoxia-inducible factors (HIFs) play an essential role in determining trophoblast fate [52, 53]. Hypoxia-induced HIF-1α expression was hindered by *ENPEP* deprivation in murine aortic endothelial cells [54]. We also found an upregulation of HIF-1α expression during trophoblast differentiation from hEPSC [55]. Thus, ENPEP might control trophoblast differentiation by regulating the HIF-1α pathway. The delayed reduction of *OCT4* in the *ENPEP*-KO trophoblastic spheroids may be associated with a blunted HIF-1α signaling, as HIF-1α could suppress *Oct4* expression via direct binding to the reverse hypoxia-responsive elements in the *Oct4* promoter [56].

ENPEP is known as a glutamyl aminopeptidase which cleaves the N-terminal aspartic acid of angiotensin (ANG) II [57]. Human placenta can synthesize ANG II [58, 59]. Notably, ANG II decreases expression and secretion of E-cadherin in EEC [60] and downregulates the production of invasion-related proteolytic enzyme, plasmin in trophoblasts [61, 62]. The absence of ENPEP might result in accumulation of ANG II at the interface of trophoblastic spheroids and EEC, thereby reducing the E-cadherin mediated attachment process and trophoblast invasion as observed in the *ENPEP*-null trophoblastic spheroids. Furthermore, TSCs derived from *ENPEP*-KO hEPSC-em failed to differentiate into STB properly, but they could still form EVT-like cells, suggesting ENPEP might be dispensable for early EVT differentiation.

We next attempted to delineate the roles of TET proteins in epigenetic regulation of *ENPEP* during early trophoblast differentiation. Our ChIP-qPCR results demonstrated a prominent recruitment of TET2 protein, rather than TET1 and TET3, at the *ENPEP* promoter during initiation of trophoblast differentiation in trophoblastic spheroids at 24 h. The reduced TET2 bindings at the *ENPEP* promoter in trophoblastic spheroids at 48 h might allow exposure of newly unmethylated DNAs and facilitate their subsequent transcriptions [63, 64]. These results also explained the findings that DMOG treatment had the highest effect at 0–24 h but not 24–48 h in delaying the reduction of DNA methylation level in the *ENPEP* promoter and suppression of its expression. Since the transition from the morula-like stage to TE-like stage occurred from trophoblastic spheroids at 24 h to 48 h [23], the TET2-mediated loss of DNA methylation in *ENPEP* promoter at 24–48 h may reinforce the trophoblast fate during this process. The involvement of TET2 in regulating ENPEP was further supported by the findings that TET2 deletion significantly decreased *ENPEP* expression during trophoblast differentiation.

TETs are methylcytosine dioxygenases involved in successive oxidation and conversion of 5mC to 5hmC, 5fC, and 5caC [65, 66]. It was reported that TET2 preferentially bound to 5mC over 5hmC [67], which poses a challenge for bisulfite sequencing since it cannot differentiate between the two [68]. As the oxidized methylcytosines are not recognized by DNMT1 [69], the loss of methylation in *ENPEP* promoter might be due to TET2 initiated replication-dependent passive dilution [70] or thymine DNA glycosylase (TDG)-dependent active demethylation [65, 71]. Further examination is required to confirm if there exist other molecules affecting the methylation status of *ENPEP* promoter during trophoblast formation. The function of TET2 has been widely studied in hematopoiesis and immune regulation [72, 73]. Whether TET2 is critical in regulating early human trophoblast differentiation and the underlying mechanisms remain largely unknown. It was reported that Tet2 deletion impaired the stem cell state of mouse TSC, with reduced TSC marker expressions and loss of epithelial characteristics [74]. In human, downregulation of TET2 expression was detected in placenta from the preeclampsia patients and was related to the reduced trophoblast migration and invasion abilities [75]. In this study, we also found that the *TET2* null hEPSC-em exhibited attenuation on trophoblast differentiation and TSC formation ability, indicating the critical roles of TET2 in governing early trophoblast differentiation. The alternative targets of TET2 or other epigenetically regulated molecules during early trophoblast differentiation warrant further investigation.

In summary, this study highlighted the crucial roles of loss of DNA methylation in prompting early trophoblast differentiation. More importantly, TET2-mediated loss of DNA methylation in *ENPEP* promoter played a vital role in trophoblast fate commitment.

### Supplementary Information

Below is the link to the electronic supplementary material.

Supplementary file1 (JPG 888 KB)

Supplementary file2 (JPG 309 KB)

Supplementary file3 (JPG 439 KB)

Supplementary file4 (JPG 1056 KB)

Supplementary file5 (JPG 633 KB)

Supplementary file6 (PDF 177 KB)

## Data Availability

This study includes no data deposited in external repositories. The data that support the findings of this study are available from the corresponding author upon reasonable request.
