# Supplementary figures and images for: Uncovering the role of TET2-mediated ENPEP activation in trophoblast cell fate determination
Source: Cell Mol Life Sci. 2024 Jun 17;81(1):270. doi: 10.1007/s00018-024-05306-z (PMC11335190; doi:10.1007/s00018-024-05306-z)

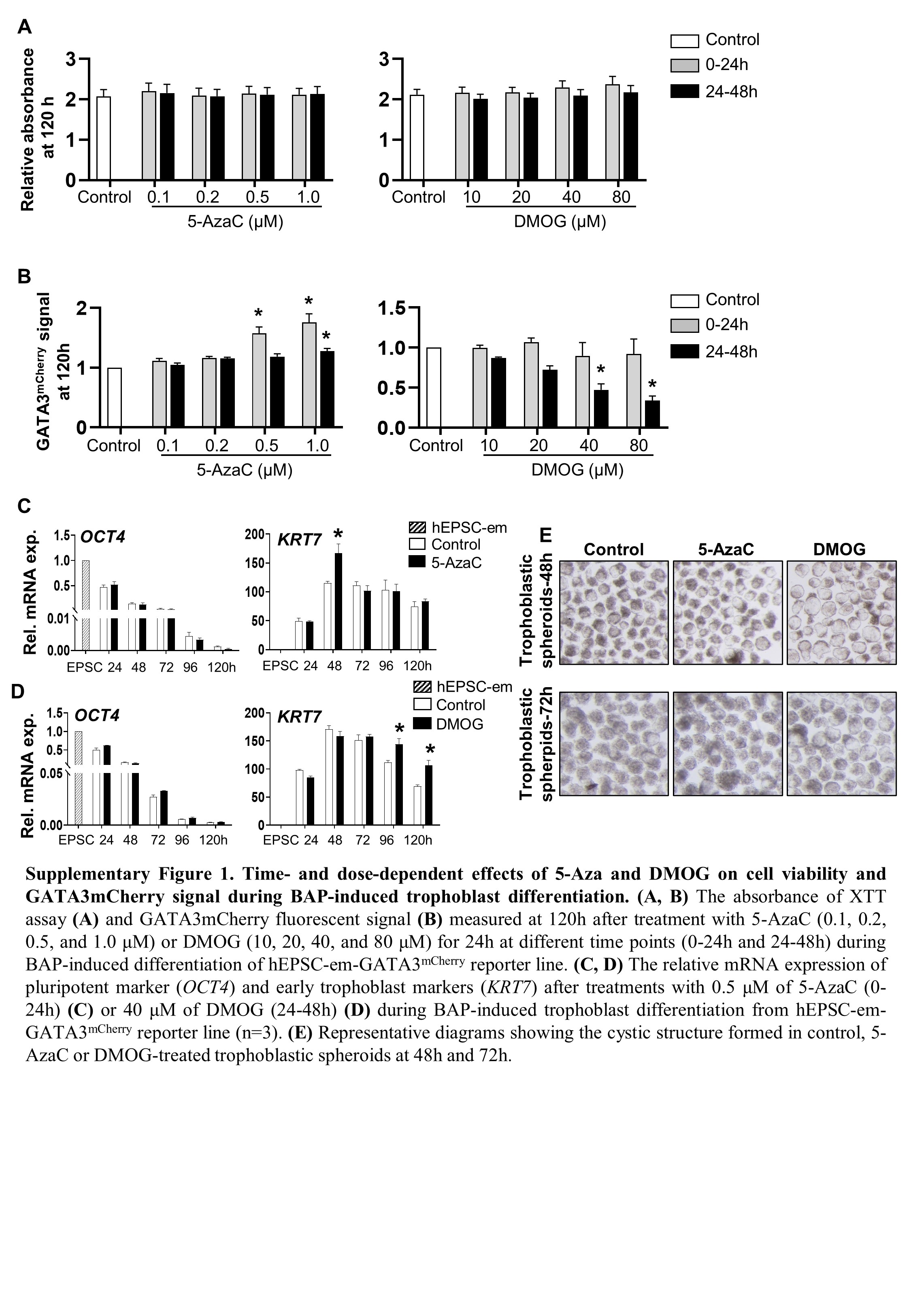

Supplement: Supplementary file 1 — Supplementary file1 (JPG 888 KB) [file 18_2024_5306_MOESM1_ESM.jpg]

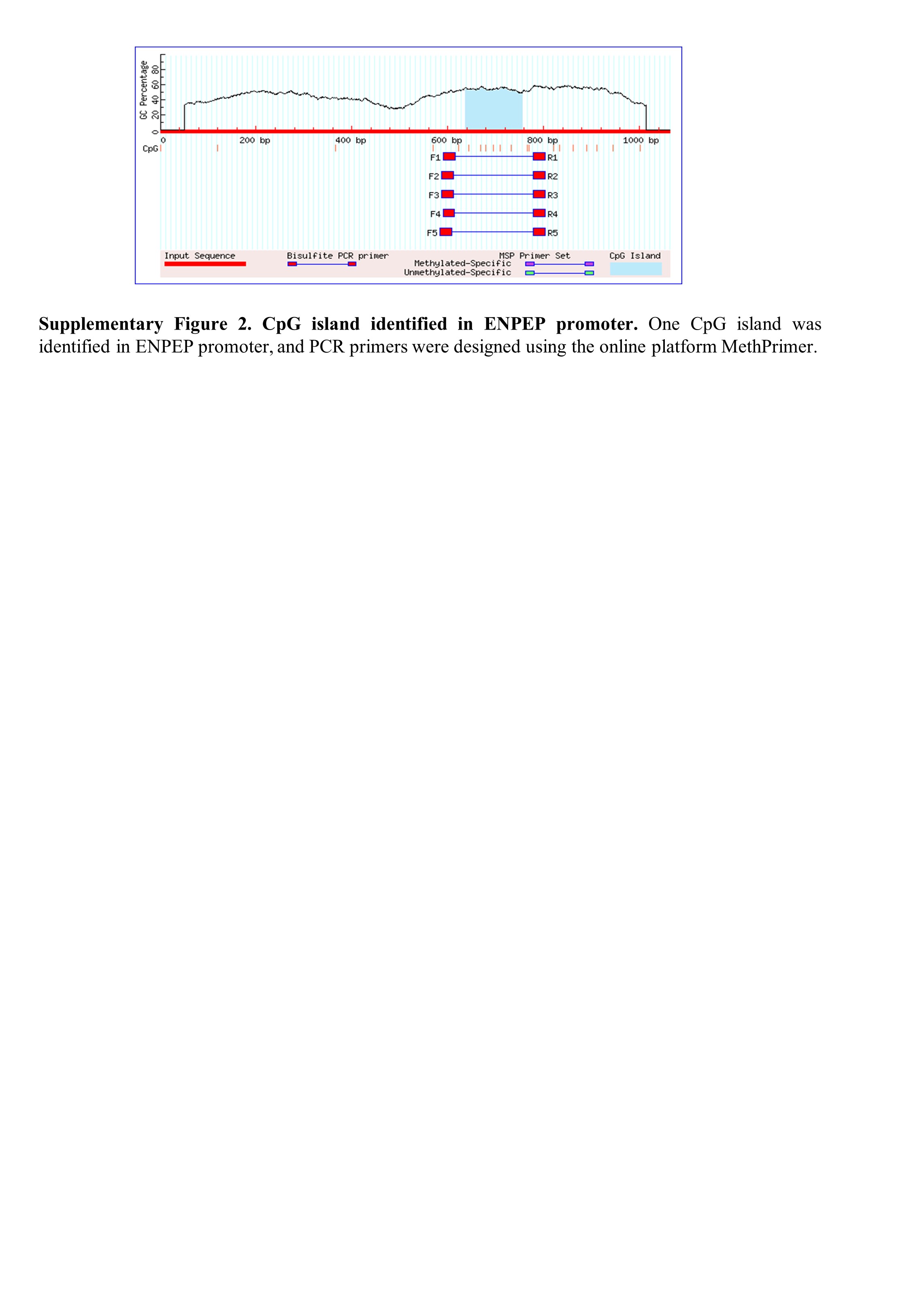

Supplement: Supplementary file 2 — Supplementary file2 (JPG 309 KB) [file 18_2024_5306_MOESM2_ESM.jpg]

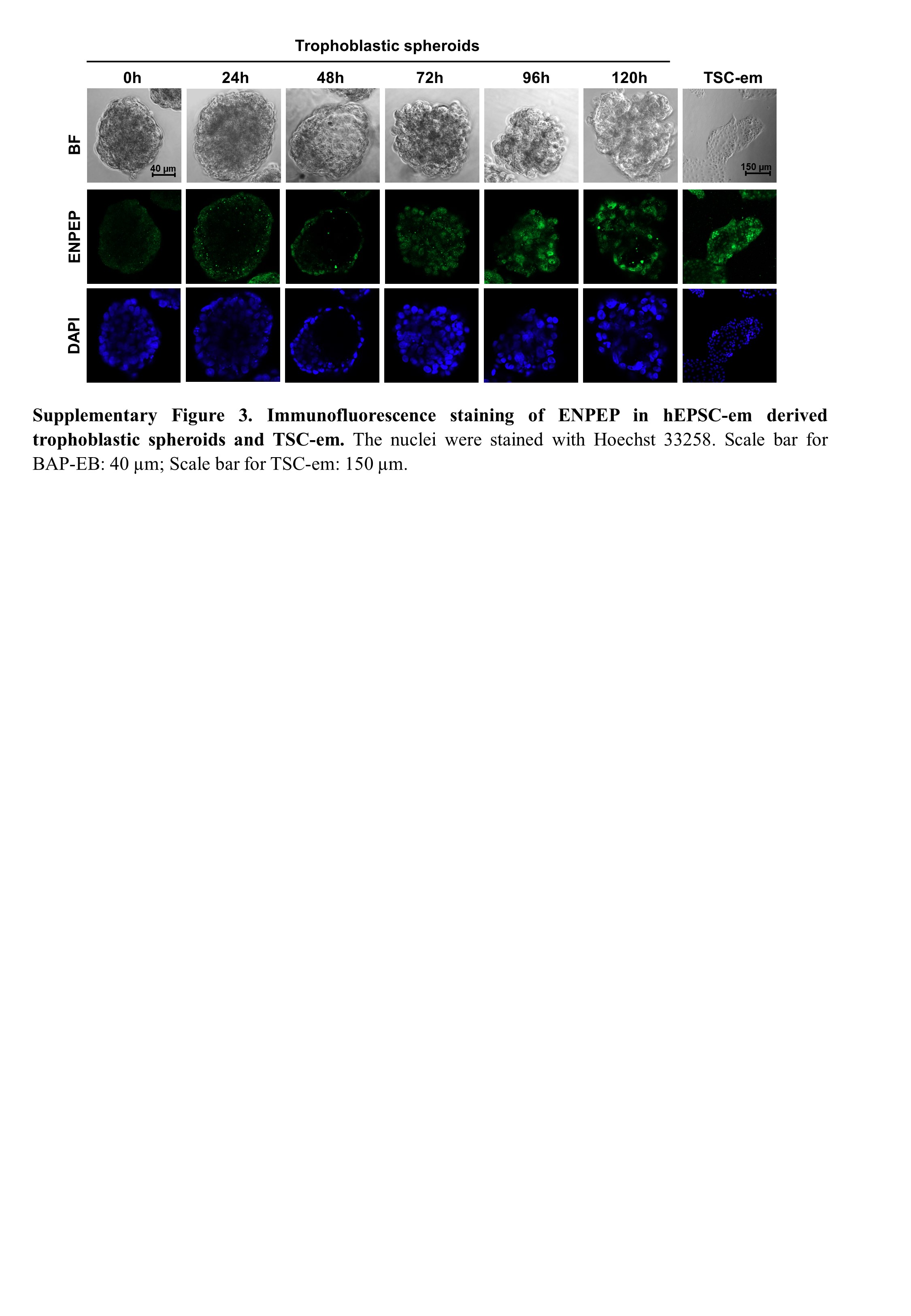

Supplement: Supplementary file 3 — Supplementary file3 (JPG 439 KB) [file 18_2024_5306_MOESM3_ESM.jpg]

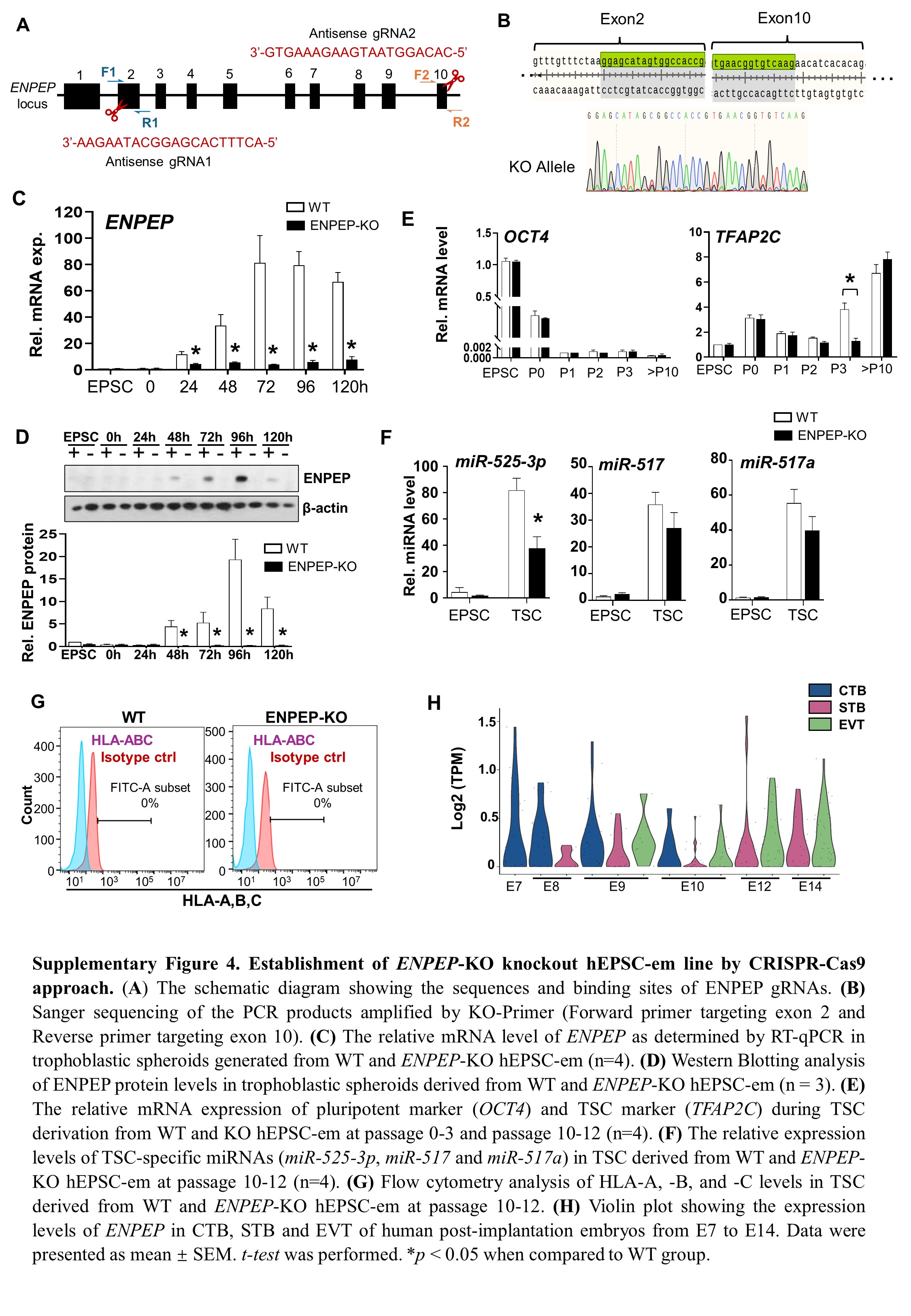

Supplement: Supplementary file 4 — Supplementary file4 (JPG 1056 KB) [file 18_2024_5306_MOESM4_ESM.jpg]

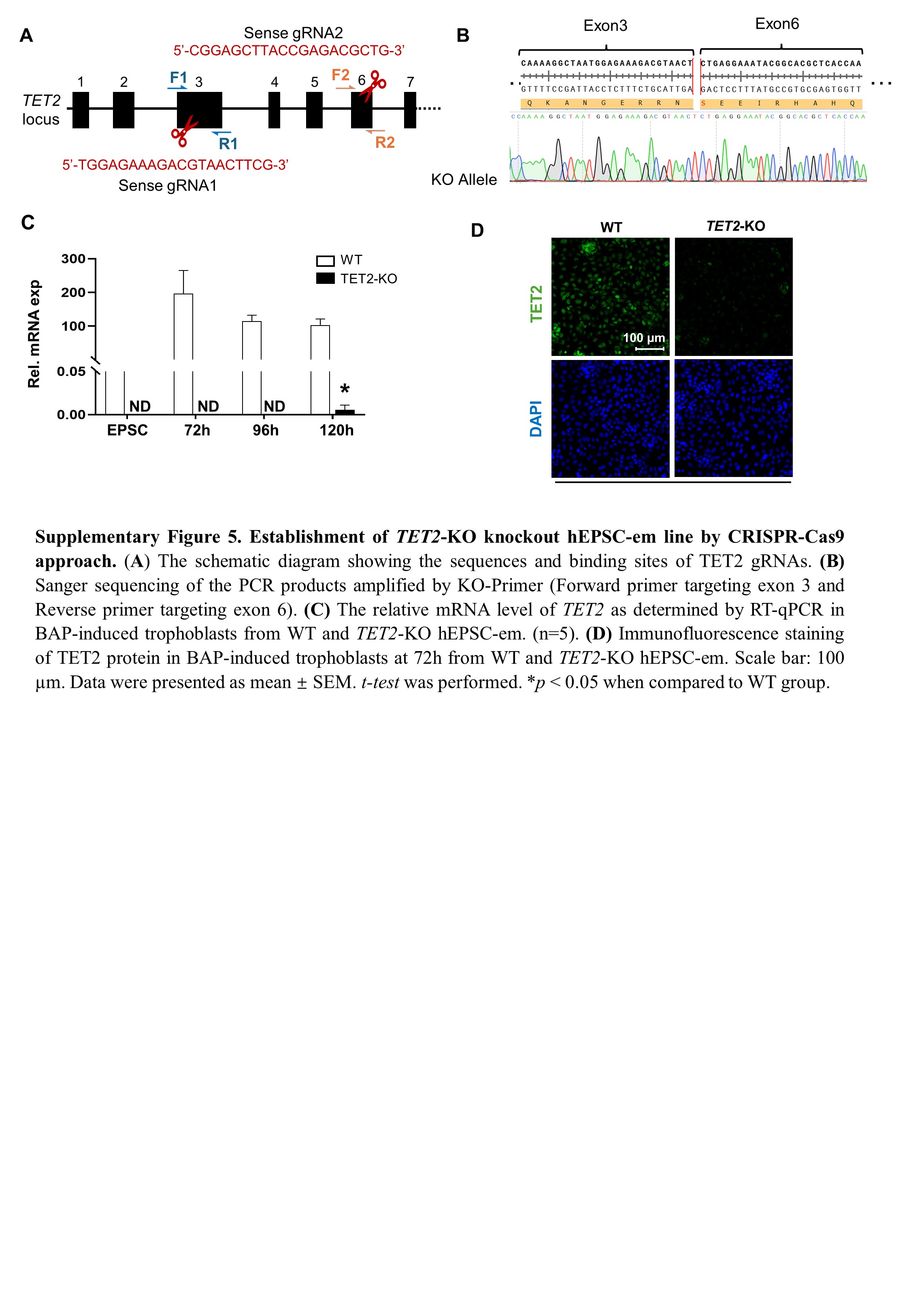

Supplement: Supplementary file 5 — Supplementary file5 (JPG 633 KB) [file 18_2024_5306_MOESM5_ESM.jpg]
