# Supplementary material for: Uncovering the role of TET2-mediated ENPEP activation in trophoblast cell fate determination
Source: Cell Mol Life Sci. 2024 Jun 17;81(1):270. doi: 10.1007/s00018-024-05306-z (PMC11335190; doi:10.1007/s00018-024-05306-z)
Supplement: Supplementary file 6 — Supplementary file6 (PDF 177 KB) [file 18_2024_5306_MOESM6_ESM.pdf]

Supplementary information

Table S1. PCR primers

Gene	Location	Primer sequences	Tm (°C)
ENPEP	Exon 2	Forward: CAGCTGTTCACTTCCCATAA	59.1
		Reverse: CACTTCCCACTCACCAAAG	
	Exon 10	Forward: GGACCAGACAGATGGGTTATC	59.1
		Reverse: CAGCCTCACTTCTCACTCTTAC	
	Promoter	Forward: TTTAGGTTGAGTGGTAAAGGTTGAG	58.3
		Reverse: TACAAAAAAATTATCACAACTCCCC	
TET2			
	Exon 3	Forward: TGGAGAAAGACGTAACTTCG	55.8
		Reverse: TGGAGAAAGACGTAACTTCG	
	Exon 6	Forward: GTCCATTCTAGTGCCTGCTAAA	55.8
		Reverse: AGACACTCTGACTGCTCCTAA	

Table S2. Antibodies

Antibody	Catalog number	Host	Application	Concentration/Dilution
Anti-TET1	GTX124207 (GeneTex)	Rabbit	ChIP	1.05 mg/mL
Anti-TET2	A304-247A (Bethyl Laboratories)	Rabbit	ChIP, ICC	1 mg/mL, 1:200
Anti-TET3	GTX121453 (GeneTex)	Rabbit	ChIP	1 mg/mL
Anti-ENPEP	MA1028 (Invitrogen)	Mouse	WB	1:1000
Anti- β-actin	A5316 (Sigma- Aldrich)	Mouse	WB	1:10000
Anti-ENPEP	MA5-25787(Invitrogen)	Mouse	ICC	1:100

Table S3. TaqMan qPCR Primers

Gene symbols	Assay ID
POU5F1(OCT4)	Hs00999632_g1
GATA2	Hs00231119_m1
GATA3	Hs00231122_m1
KRT7	Hs00559840_m
TFAP2C	Hs00231476_m1
CGB3	Hs00361224_gH
ERVW-1	Hs00205893_m1
SDC1	Hs04966523_m1
GCM1	Hs00172692_m1
HLA-G	Hs00365950_g1
MMP2	Hs01548727_m1
ENPEP	Hs00989749_m1
DNMT1	Hs00945875_m1
DNMT3A	Hs01027162_m1
DNMT3B	Hs00171876_m1
DNMT3L	Hs01081364_m1
TET1	Hs04189344_g1
TET2	Hs00325999_m1
TET3	Hs00896441_m1
has-miR-103	000439
hsa-miR-525b-3p	002385
has-miR-517a	002402
has-miR-517	001113

Table S4. Primer sequences for ChIP-qPCR

Primer	Sequences
ENPEP-1	Forward: CACCCAATTCCAGGATATGAC
	Reverse: GCAATCTCTCCCTCCTAACTA

ENPEP-2	Forward: GGGTGGAGATGCGTCGTTTA
	Reverse: GCTTCTTGCGCCTTTTCCTC
ENPEP-3	Forward: CCTCCCGTTGGCTGATA
	Reverse: GGAATGAGGACTCCGTAAGA
